# Role of antifungal drugs in modulating interaction between *Candida glabrata* and macrophages: cytokine profiles and TEM analysis

**DOI:** 10.1128/spectrum.00872-25

**Published:** 2025-10-22

**Authors:** Xinyi Wang, Zhenghui Yang, Yuye Li, Tianxiang Dong, Yi-Qun Kuang, Hongbin Li

**Affiliations:** 1 Department of Dermatology and Venereology, First Afﬁliated Hospital of Kunming Medical University, Kunming, China; 2 Research Center for Clinical Medicine, First Afﬁliated Hospital of Kunming Medical University, Kunming Medical University, Kunming, China; 3 Yunnan Key Laboratory of Stem Cell and Regenerative Medicine, Kunming Medical University 71240, Kunming, China; Mayo Foundation for Medical Education and Research, Rochester, Minnesota, USA

**Keywords:** *Candida glabrata*, macrophage, immune evasion, cytokines, transmission electron microscopy (TEM), itraconazole, micafungin, amphotericin B

## Abstract

*Candida glabrata* often resides within macrophages, creating challenges for antifungal treatment. This study investigates the impact of antifungal agents on the immune response of *C. glabrata* within macrophages to understand its immune evasion mechanisms. Standard *C. glabrata* strains ATCC2001 and a clinical strain (17K1152) were pretreated with micafungin (MCF), itraconazole (ICZ), and amphotericin B (AmB) for 24 hours and then co-cultured with macrophages for 6, 12, 24, and 48 hours. Transmission electron microscopy (TEM) analysis was performed, and cytokine levels (interleukin [IL]-6, granulocyte-macrophage colony-stimulating factor [GM-CSF]) were measured using enzyme-linked immunosorbent assay kits. *C. glabrata* showed the greatest susceptibility to MCF, followed by AmB, with ICZ having the least effect. TEM analysis revealed MCF-induced damage to both the cell wall and membrane, whereas AmB disrupted only the membrane. ICZ showed less noticeable damage. MCF-pretreated yeast cells were more readily damaged in macrophages than those pretreated with AmB or ICZ. IL-6 secretion patterns were similar in untreated and ICZ-treated groups, while AmB initially decreased IL-6 secretion before recovery, and MCF consistently increased IL-6 levels. GM-CSF secretion showed comparable trends for untreated and ICZ groups, with MCF and AmB exhibiting fluctuations. Echinocandins enhanced macrophage immune activity by modifying *C. glabrata*’s cell wall and increasing pro-inflammatory cytokine secretion, impairing its survival within host cells. Azole pretreatment had minimal impact on yeast replication and survival, reflecting resistance. Polyenes-treated yeast evade innate immunity by reducing pro-inflammatory cytokine secretion and modulating macrophage recruitment.

**IMPORTANCE:**

*Candida glabrata* is a major cause of bloodstream infections and is often resistant to antifungal treatment. Understanding how different antifungal drugs affect its survival within macrophages is critical for improving therapy. This study demonstrates that echinocandins enhance macrophage-mediated killing by damaging the fungal cell wall and increasing pro-inflammatory cytokine secretion, whereas azoles have minimal effect, and polyenes may aid immune evasion. These findings provide mechanistic insights into antifungal resistance and host-pathogen interactions, informing strategies for more effective treatment of *C. glabrata* infections.

## INTRODUCTION

*Candida* species are the leading cause of invasive fungal infections (IFI) and rank as the fourth most common cause of nosocomial bloodstream infections (1–3). In recent years, non-*albicans Candida* infections have notably increased. The rising incidence of *Candida* infections may be attributed to an increasing number of immunocompromised patients (e.g., those with AIDS or organ transplants), the aging population, advancements in catheter technology, and the widespread use of azole antifungals (4). In Northern Europe and the United States, *Candida glabrata* has become the second most common pathogen following *Candida albicans*, with a mortality rate of approximately 40% (5–8). In Asia, *C. glabrata* ranks as the third or fourth most common pathogenic species (9, 10). However, our previous research indicated that *C. glabrata* was the second most common pathogen of IFI in general hospitals, following *C. albicans* (11).

The resistance of *C. glabrata* had made it a significant clinical concern, with its immune evasion strategies playing a key role in causing systemic infections. Unlike *C. albicans*, *C. glabrata* exists in a spore form and does not form hyphae for tissue invasion. Studies indicate that its invasive mechanisms rely on various virulence factors, including adhesion, biofilm formation, and hydrolytic enzyme activity (12–14). Additionally, *C. glabrata* may enhance its invasive capacity through induced host cell endocytosis (15). Successful evading of host defenses is essential for *C. glabrata* to establish, invade, and disseminate within the host, contributing to persistent infections and treatment resistance.

The host mucosa acts as the first barrier to prevent *C. glabrata* dissemination. Upon breaching this barrier, *C. glabrata* encounters innate immune cells, such as macrophages, which activate the host’s innate immune response. Macrophages are crucial to innate immunity, as they recognize, engulf, and degrade pathogens like *Candida*, while secreting pro-inflammatory and anti-inflammatory cytokines and chemokines to initiate adaptive immunity. These responses are generally effective at eliminating most phagocytosed microorganisms. However, *C. glabrata* is not only highly resistant to antifungal drugs but also capable of adapting and evading immune defenses, allowing it to survive and disseminate within hosts (16). Successful evasion of macrophage-mediated killing is therefore pivotal for *C. glabrata* (17). If *C. glabrata* is killed post-phagocytosis, dissemination is prevented. However, if *C. glabrata* resists phagocytosis and survives, it may enter the bloodstream, leading to disseminated infections.

A recent study found that pretreating macrophages with three antifungal agents revealed that micafungin reduced pro-inflammatory cytokine secreted by macrophages compared to itraconazole and amphotericin B. The authors proposed that echinocandins might enhance antifungal efficacy of macrophages by modulating macrophage responses (18). However, no studies have examined the impact of antifungal pretreatment of *C. glabrata* on its interactions within macrophages, and the mechanisms by which antifungal agents influence *Candida* evasion from macrophage killing remain unclear. Clinically used antifungal agents for IFI include azoles (e.g., fluconazole, itraconazole, voriconazole, and posaconazole), echinocandins (e.g., caspofungin, micafungin, and anidulafungin), polyenes (e.g., amphotericin B and nystatin), and flucytosine (e.g., 5-fluorocytosine). Due to the high resistance of *C. glabrata* to azoles and the potential for it to develop resistance to other antifungal agents, including multidrug resistance, resistance rates are rising annually, highlighting an urgent need for new antifungal intervention.

This study employed transmission electron microscopy (TEM) and measurement of pro-inflammatory cytokines (interleukin [IL]-6, granulocyte-macrophage colony-stimulating factor [GM-CSF]) at various time points after macrophages with *C. glabrata* pretreated with three commonly used antifungal agents (itraconazole, micafungin, and amphotericin B) as well as untreated controls. These analyses aim to clarify the impact of antifungal agents on *C. glabrata* immune evasion mechanisms, providing valuable insights into the relationship between *C. glabrata* pathogenicity and antifungal treatments and informing more effective strategies for the prevention and treatment of candidiasis.

## MATERIALS AND METHODS

### Reagents

Dimethyl sulfoxide (Beijing Solabao Technology Co., Ltd.), AlamarBlue (Beijing Solabao Technology Co., Ltd.), glucose powder (OXOID, United Kingdom), agar powder (Beijing Solabao Technology Co., Ltd.), yeast extract (Beijing Solabao Technology Co., Ltd.), peptone (OXOID, United Kingdom), IL-6 ELISA kit (Jiangsu Meimian Industrial Co., Ltd.), GM-CSF ELISA kit (Jiangsu Meimian Industrial Co., Ltd.), fetal bovine serum BC-SE-FBS08 (Nanjing Senbei Jia Biological Technology Co., Ltd.), 0.01M phosphate buffer (pH 7.4), Dulbecco's Modified Eagle Medium (DMEM) basic (1×) (Thermo Fisher Scientific Inc.), electron microscopy fixative (Servicebio), anhydrous ethanol (China National Pharmaceutical Group Corporation), acetone (China National Pharmaceutical Group Corporation), epoxy resin embedding medium 812 (SPI Supplies), osmium tetroxide (Ted Pella Inc.), penicillin-streptomycin mixed solution (100×) (Biosharp), amphotericin B deoxycholate (Beijing Solabao Technology Co., Ltd.), miconazole (Shanghai Yuanye Biological Technology Co., Ltd.), and itraconazole (Beijing Solabao Technology Co., Ltd.).

### Fungal strains

The *C. glabrata* standard strain ATCC2001 was purchased from the American Type Culture Collection (ATCC). The clinical isolate *C. glabrata* 17K1152 was obtained from a peritoneal drainage fluid specimen cultured by the Laboratory Department of the First Affiliated Hospital of Kunming Medical University. Both strains were identified as *C. glabrata* by ITS sequencing and database comparison (blast.ncbi.nlm.nih.gov) with accession of National Center for Biotechnology Information registration numbers as KU052054.1 for 17K1152 and KP674677.1 for ATCC2001.

### Propagation and preparation of yeast suspensions

Using sterile disposable inoculating loops, the fungal strains (ATCC2001 and 17K1152) were inoculated onto a yeast extract–peptone–dextrose (YPD) agar medium and incubated in a 35°C incubator for 3–5 days until mature yeast colonies were formed. Subsequently, a single colony was picked using a sterile inoculating loop and inoculated into 2 mL of YPD broth medium and grown overnight at 35°C in a shaking incubator at 150 rpm.

### MIC Determination

Antifungal susceptibility testing for *C. glabrata* strains ATCC 2001 and 17K1152 was performed using the broth microdilution method recommended by the Clinical and Laboratory Standards Institute (CLSI) document M27-A3. RPMI-1640 medium buffered with 3-(N-morpholino)propanesulfonic acid was used as the testing medium, and assays were carried out in sterile, flat-bottom 96-well microtiter plates. Stock solutions of micafungin, itraconazole, and amphotericin B were prepared according to the manufacturers’ specifications and serially diluted in RPMI-1640 medium to achieve the desired concentrations.

Yeast inocula were prepared to a final concentration of 0.5 × 10³ to 2.5 × 10³ CFU/mL and added to the wells together with the antifungal agents. Alamar Blue reagent was used as a redox indicator to facilitate visual minimum inhibitory concentration (MIC) endpoint determination. Plates were incubated at 37°C for 24 hours.

The MIC for each antifungal was defined as the lowest drug concentration at which a visible color change from blue to pink was inhibited, indicating significant growth inhibition relative to the growth control. MICs were interpreted according to the criteria described in CLSI documents M27 and M57, and the results were validated using both positive (no drug) and negative (medium only) control wells.

### Antifungal drug pretreatment of fungi

Stock solutions of itraconazole, micafungin, and amphotericin B were diluted to concentrations corresponding to the MIC values of strains ATCC 2001 and 17K1152. Yeast suspensions of ATCC 2001 and 17K1152 were centrifuged at 1,500 rpm for 5 minutes, washed three times with sterile phosphate buffered saline (PBS) (pH 7.4), and centrifuged again to collect the yeast pellets.

For the experimental group, antifungal drug solutions at 1 × MIC concentration were added to the fungal suspensions, which were adjusted to a cell density corresponding to their MIC values. For the control group, drug-free YPD medium was added to the fungal suspensions. All mixtures were resuspended by pipetting and incubated at 37°C for 24 hours.

### Macrophage cell culture and infection with *C. glabrata*

The RAW264.7 cell line, a murine monocyte-macrophage leukemia cell line derived from adult male BALB/c mouse and characterized by adherent growth, was obtained from Procell Life Science & Technology Co., Ltd. Cells, which were nearly round in shape, were cultured in complete medium consisting of high-glucose DMEM, 5% fetal bovine serum, and 1% penicillin-streptomycin. The cultures were remained in an incubator at 37°C with 5% CO_2_ and seeded into 6-well plates at a density of 1 × 10^7^ cells per well. The plates were incubated overnight until the cells were fully adherent, followed by infection with *C. glabrata* in fresh medium.

To ensure that residual antifungal drugs did not interfere with the interaction between *C. glabrata* and macrophages, the pretreated ATCC2001 and 17K1152 yeast suspensions were centrifuged at 1,500 rpm for 5 minutes to completely remove the old medium containing antifungal drugs. The yeast cells were then washed twice with sterile PBS (pH 7.4) to eliminate any remaining drug traces. After washing, the yeast cells were resuspended in complete medium, manually counted, and diluted to an appropriate concentration. Macrophages were infected with *C. glabrata* at a multiplicity of infection of 2 and incubated at 37°C with 5% CO_2_. At designated time points post-infection (2, 6, 12, 24, and 48 hours), supernatants were collected, centrifuged to remove debris, and stored at −80°C. Additionally, at 6, 24, and 48 hours, electron microscopy fixative was added to the cells, which were then stored at 4°C.

### Determination of cytokine concentrations (IL-6, GM-CSF)

Assay kits were removed from refrigeration and equilibrated to room temperature for 1 hour. Cell supernatants were retrieved from −80°C storage and thawed. For the assay, washing buffer and standard solutions for IL-6 and GM-CSF were prepared. The procedure involved adding samples or reagents to the blank, standard, and sample wells on the ELISA plate, followed by incubation, washing, addition of enzyme, a second incubation, further washing, color development, and stopping the reaction. The cytokine concentrations were determined by plotting standard curves to calculate the concentrations of the samples.

### TEM of macrophages phagocytosing *C. glabrata*

Cell pellets were collected and fixed in electron microscopy fixative at 4°C for preservation and transport. The cells were then prepared for embedding by washing, centrifuging, and suspending in an agarose solution. Post-fixation was performed using osmium tetroxide. Following post-fixation, samples were dehydrated at room temperature through a graded series of ethanol and acetone. For infiltration and embedding, samples were placed in epoxy resin embedding medium 812 and left to infiltrate overnight. The samples were then polymerized, sectioned into ultrathin sections, and stained with uranyl acetate and lead citrate. Stained sections were examined under a transmission electron microscope (Hitachi HT7700), and images were captured for analysis.

### Statistical methods

Statistical analysis was conducted with SPSS 26 software. Intergroup comparisons were performed using two-way analysis of variance, followed by multiple comparisons, with *P < 0.05* considered statistically significant. Data visualization was completed using GraphPad Prism 9.0 software. All key experiments were independently repeated at least three times with consistent results.

## RESULTS

### *C. glabrata* exhibits the highest susceptibility to echinocandins and a high level of resistance to azoles

*C. glabrata* showed the greatest susceptibility to the echinocandin micafungin and exhibited reduced susceptibility to the azole itraconazole. As shown in Table 1, the MIC values for the 17K1152 strain were higher than those of the ATCC2001 strain across all three antifungal agents tested, indicating decreased susceptibility. Notably, the MIC for micafungin in the clinical isolate (0.125 µg/ml) falls within the intermediate susceptibility range according to the CLSI M27 document. Although there are no established clinical breakpoints for itraconazole and amphotericin B against *C. glabrata*, the MICs observed for the clinical isolate (4 µg/mL for itraconazole and 2 µg/mL for amphotericin B) correspond to the published epidemiological cutoff values per the CLSI M57 document, suggesting the presence of non-wild-type characteristics.

**TABLE 1:** MIC values (µg/mL) of three antifungal agents against standard and clinical strains of *C. glabrata*

Strain	Micafungin	Itraconazole	Amphotericin B
ATCC2001	0.0625	1	0.5
17K1152	0.125	4	2

### Morphological and dynamic difference in phagocytosis between two *C. glabrata* strains without antifungal intervention

**Fig 1:**
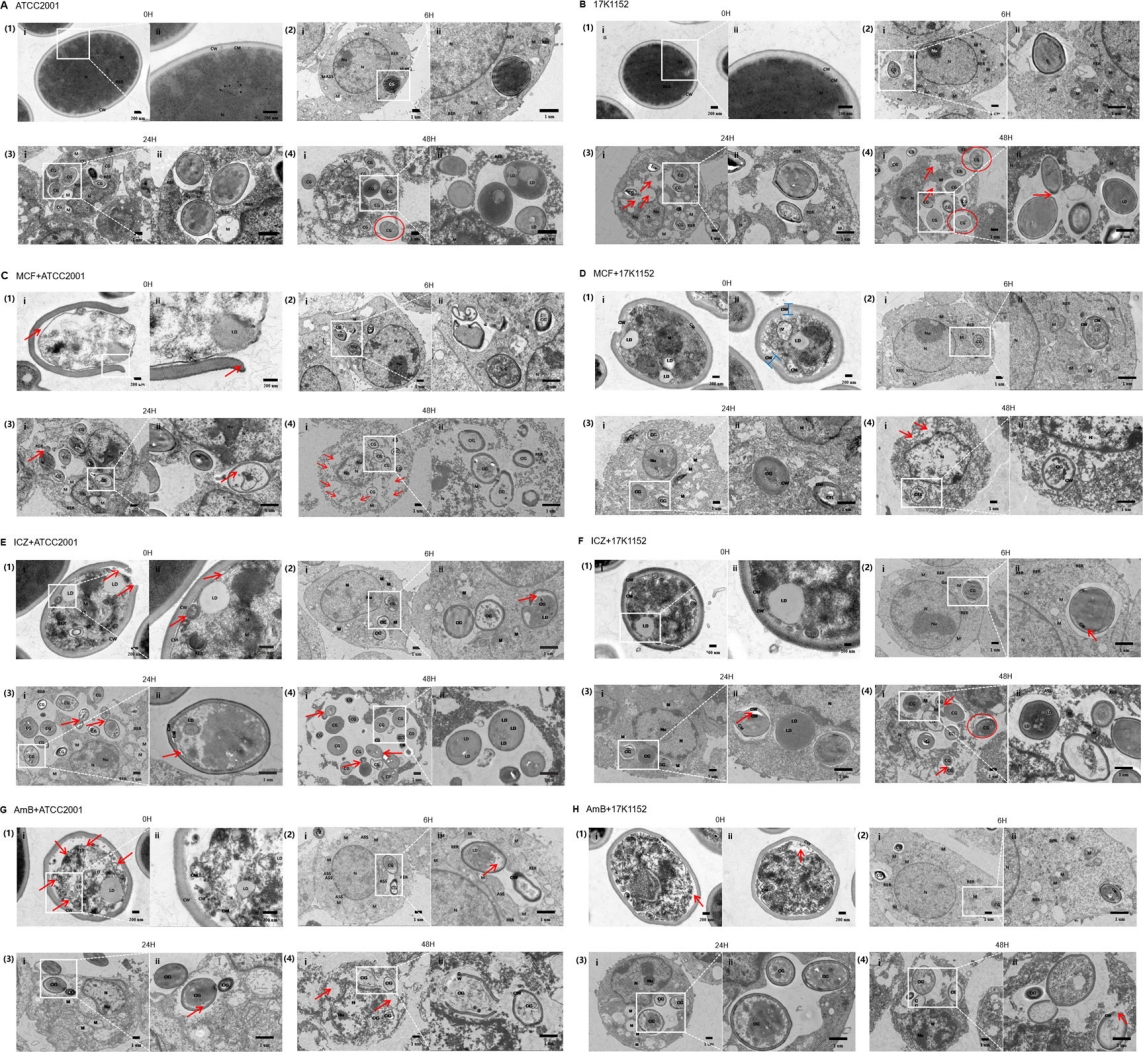
Raw264.7 macrophages phagocytosing *C. glabrata*: (**A**) untreated ATCC2001 strain; (**B**) untreated 17K1152 strain; (**C**) ATCC2001 strain pretreated with micafungin; (**D**) 17K1152 strain pretreated with micafungin; (**E**) ATCC2001 strain pretreated with itraconazole; (**F**) 17K1152 strain pretreated with itraconazole; (**G**) ATCC2001 strain pretreated with amphotericin B; (**H**) 17K1152 strain pretreated with amphotericin B. Magnifications: (i)i (×15,000), (1)ii (×30,000), (2)i, (3)i, (4)i (×3,000), (2)ii, (3)ii, (4)ii (×7,000). TEM shows the yeast structure before phagocytosis (0 hour) and the interaction between cells and yeast at different time points (6, 24, 48 hour). Symbols in the figure: cell wall (CW), cell membrane (CM), nucleus (N), nucleolus (Nu), mitochondria (M), yeast (CG), lipid droplets (LD), autophagolysosome (ASS).

Significant differences were observed between the standard and clinical strains of *C. glabrata* in macrophage phagocytosis without drug intervention. For the ATCC2001 strain, the untreated standard exhibited regular morphology with intact cell walls and membranes prior to macrophages phagocytosed (Fig. 1A(1)). Six hours post-phagocytosis, macrophages largely retained normal morphology (Fig. 1A(2)). By 24 hours, macrophages presented signs of severe mitochondrial swelling (Fig. 1A(3)), and at 48 hours, cytoplasm was sparse, organelles appeared blurred, and “exocytosis was observed (Fig. 1A(4)).

In contrast, for the 17K1152 strain, macrophages exhibited slight elongation, mild to moderate mitochondrial swelling, and visible vesicular structures by six hours post-phagocytosis (Fig. 1B(2)). After 24 hours, macrophage membranes showed slight damage, with sparse cytoplasm and vacuolated organelles; yeast cytoplasm appeared slightly lighter (Fig. 1B(3)). By 48 hours, most macrophage membranes were damaged, “exocytosis” was prominent, and yeast cells had increased in size, with some cells in the budding stage and a few containing lipid droplets (Fig. 1B(4)).

Overall, the ATCC2001 strain caused more severe damage to macrophages, while the 17K1152 strain showed more active exocytosis and yeast proliferation.

### Antifungal drug pretreatment induced morphological changes of macrophages and *C. glabrata*

Following the phagocytosis of *C. glabrata* by macrophages, the degree of macrophage damage progressively increased over time. Six hours post-phagocytosis, the macrophage morphology and organelles remained largely intact, with one to three yeast cells observed within macrophages, all showing some degree of damage (Fig. 1(2)). By 24 hours, macrophages exhibited morphological changes, notably severe mitochondrial swelling, and in some cases, dissolution. The number of internalized yeast cells increased, particularly in the itraconazole pretreated group (Fig. 1(3)). Despite antifungal pretreatment, the internalized yeast cells exhibited varying levels of damage. At 48 hours, macrophages were partially destroyed, with variable numbers of internalized yeast cells. Like the untreated group, the yeast cells in the itraconazole group remained largely intact (Fig. 1(4)), some internalized yeast cells began budding and proliferating (Fig. 1E and F). In contrast, yeast cells in the micafungin and amphotericin B groups exhibited significantly reduced numbers within macrophages, showing edema, fragmentation, discontinuity, and, in some cases, complete dissolution of cytoplasmic contents (Fig. 1C, D, G and H).

Each of three antifungal drugs caused different levels of damage to the cell walls and membranes of the ATCC2001 strain, while the 17K1152 strain experienced considerably less damage. Micafungin primarily affected the yeast cell wall, leading to swelling, discontinuity, and separation between the cell wall and membrane, with a noticeably wider gap in the ATCC2001 strain compared to the 17K1152 strain (Fig. 1C and D). Amphotericin B mainly damaged the yeast cell membrane, causing varying degrees of shrinkage, invagination, and dissolution, with more severe membrane damage in the ATCC2001 strain compared to the 17K1152 strain (Fig. 1G and H). Itraconazole caused localized wall breakage, mild invagination of the cell membrane, and slight separation between the cell wall and membrane in the ATCC2001 strain, with minimal damage observed in the 17K1152 strain (Fig. 1E and F).

### Micafungin intervention enhances *C. glabrata* destruction by macrophages

Micafungin treatment induced notable damage in the cell wall and membrane of the ATCC2001 strain, causing cytoplasmic lightening and the presence of a few lipid droplets present (Fig. 1C(1)). Six hours after being phagocytosed by macrophages, parts of the yeast cell wall were fractured, and cytoplasmic density had decreased (Fig. 1C(2)). By 24 hours, the yeast’s cytoplasm became homogeneous, with a few yeast cells undergoing budding (Fig. 1C(3)). After 48 hours, the yeast’s cell wall and cytoplasm were severely damaged (Fig. 1C(4)). Similarly, micafungin-treated 17K1152 strain displayed damaged cell walls, reduced continuity, and slight separation of the cell membrane prior to macrophage phagocytosis (Fig. 1D(1)). Six hours after phagocytosis, some yeast cell membranes ruptured (Fig. 1D(2)). By 24 hours, the yeast cytoplasm density had decreased and the cell wall fractured (Fig. 1D(3)). After 48 hours, portions of the yeast cytoplasm had dissolved (Fig. 1D(4)).

### Itraconazole intervention causes *C. glabrata* cytoplasmic edema during phagocytosis

Under itraconazole intervention, the cell wall of the *C. glabrata* ATCC2001 strain showed slight damage, while the cell membrane exhibited shrinkage prior to phagocytosis (Fig. 1E(1)). Six hours after phagocytosis, damaged yeast cell membrane and cytoplasm were evident (Fig. 1E(2)). After 24 hours, the yeast’s cell wall remained intact, with slight shrinkage of the cell membrane (Fig. 1E(3)). By 48 hours, the cell membrane of the yeast collapsed, leading to severe cytoplasmic dissolution. Lipid droplets increased within the cytoplasm, and some yeast cells began budding (Fig. 1E(4)). Similarly, the 17K1152 strain treated with itraconazole displayed comparable membrane and cytoplasmic damage after being phagocytosed by macrophages, with frequent budding observed (Fig. 1F).

### Amphotericin B intervention significantly dissolutes and damages *C. glabrata* in macrophages

Under amphotericin B intervention, the cell membrane structure of the ATCC2001 strain of *C. glabrata* collapsed and exhibited noticeably dissolution (Fig. 1G(1)). Six hours post-phagocytosis, the yeast cell walls were swollen, and signs of cytoplasmic shrinkage and dissolution were evident (Fig. 1G(2)). After 24 hours, the yeast cell membranes exhibited mild shrinkage (Fig. 1G(3)). By 48 hours, the macrophage cytoplasm had dissolved, organelles disappeared, and the yeast cell walls showed severe swelling with partial damage (Fig. 1G(4)).

For the 17K1152 strain, prior to phagocytosis under amphotericin B intervention, multiple areas of the yeast cell membrane were damaged (Fig. 1H(1)). Six hours post-phagocytosis, the yeast cell membranes were swollen and discontinuous (Fig. 1H(2)). After 24 hours, the macrophage mitochondria exhibited severe swelling (Fig. 1H(3)). By 48 hours, the number of yeasts inside the cytoplasm had increased, with some undergoing budding reproduction (Fig. 1H(4)).

### Cytokine response of infected macrophages

#### Fluctuations in IL-6 and GM-CSF secretion by macrophages infected with two *C. glabrata* strains

**Fig 2:**
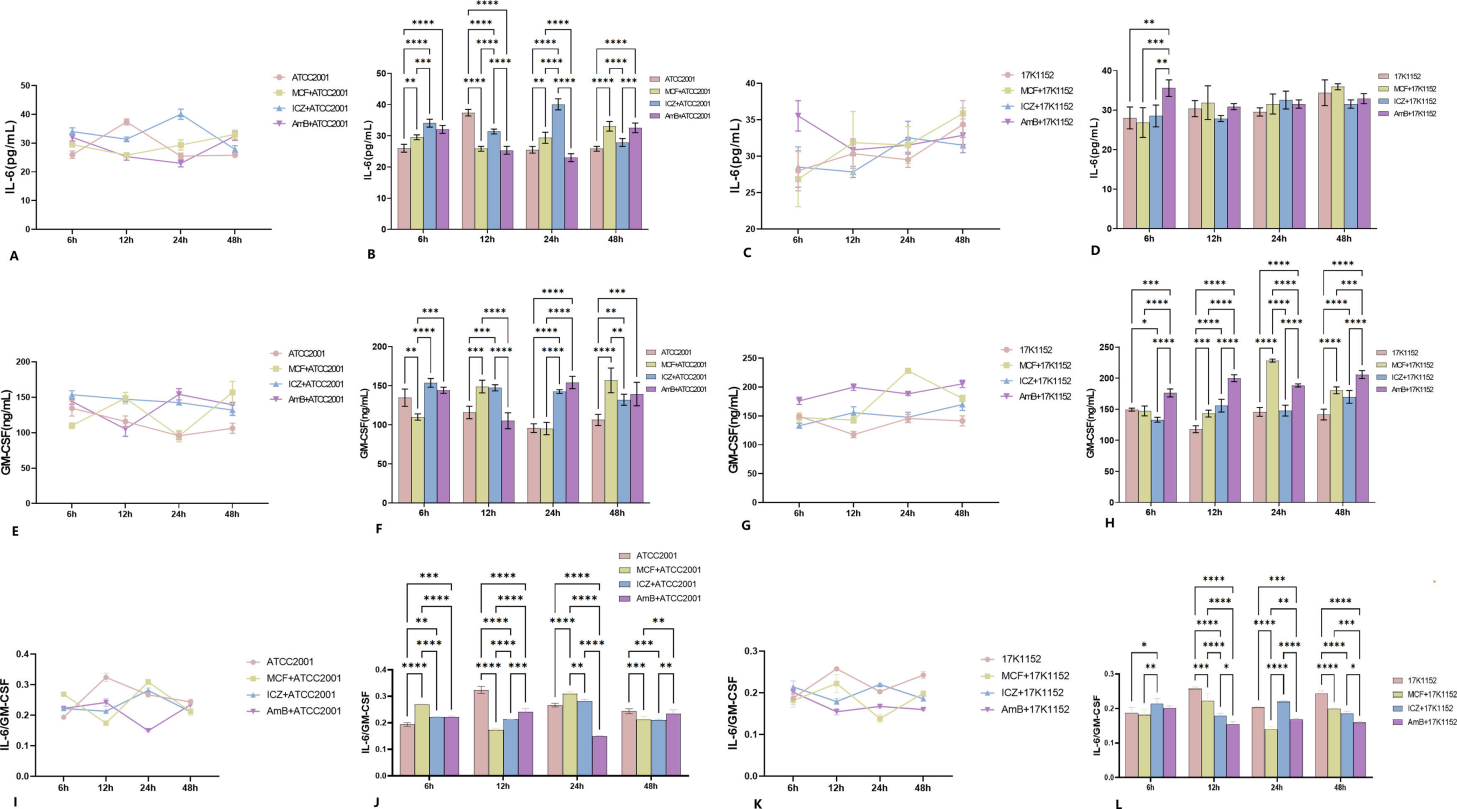
(**A**) IL-6 trends at different time points within the same group after the interaction of macrophages with the ATCC2001 strain. (**B**) Comparison of IL-6 differences between groups at the same time point after the interaction of macrophages with the ATCC2001 strain. (**C**) IL-6 trends at different time points within the same group after the interaction of macrophages with the 17K1152 strain. (**D**) Comparison of IL-6 differences between groups at the same time point after the interaction of macrophages with the 17K1152 strain. (**E**) GM-CSF trends at different time points within the same group after the interaction of macrophages with the ATCC2001 strain. (**F**) Comparison of GM-CSF differences between groups at the same time point after the interaction of macrophages with the ATCC2001 strain. (**G**) GM-CSF trends at different time points within the same group after the interaction of macrophages with the 17K1152 strain. (**H**) Comparison of GM-CSF differences between groups at the same time point after the interaction of macrophages with the 17K1152 strain. (**I**) IL-6/GM-CSF trends at different time points within the same group after the interaction of macrophages with the ATCC2001 strain. (**J**) Comparison of IL-6/GM-CSF differences between groups at the same time point after the interaction of macrophages with the ATCC2001 strain. (**K**) IL-6/GM-CSF trends at different time points within the same group after the interaction of macrophages with the 17K1152 strain. (**L**) Comparison of IL-6/GM-CSF differences between groups at the same time point after the interaction of macrophages with the 17K1152 strain. *P < 0.05; **P < 0.01; ***P < 0.001; ****P < 0.0001.

Without drug intervention, IL-6 secretion from macrophages exposed to *C. glabrata* ATCC2001 strain showed a brief increase at 12 hours (*P < 0.05*, Fig. 2A and B), followed by relatively low levels at other time points. At 12 hours, the IL-6 secretion was significantly higher compared to the drug intervention groups (Fig. 2A and B). In contrast, GM-CSF secretion in the ATCC2001 strain peaked at 6 hours, which was higher than the micafungin intervention group (*P < 0.05*, Fig. 2E and F) but lower than the itraconazole intervention group (*P < 0.05*, Fig. 2E and F). No significant difference was observed compared to the amphotericin B group (*P > 0.05*, Fig. 2E and F). The IL-6/GM-CSF ratio in the ATCC2001 strain was markedly elevated at 12 hours (*P < 0.05*, Fig. 2I and J), showing an initial rise followed by a decline.

For the 17K1152 strain, IL-6 secretion slightly increased and then declined before rising again, with the highest secretion at 48 hours; however, this was not significantly different from other groups (Fig. 2C and D). GM-CSF levels in the 17K1152 strain showed no significant changes, except for a brief drop at 12 hours (*P > 0.05*, Fig. 2G and H). Notably, the IL-6/GM-CSF levels at 12 and 48 hours were significantly elevated (*P < 0.05*, Fig. 2K and L).

#### Antifungal treatment on IL-6 and GM-CSF secretion by infected macrophages

Under micafungin intervention, IL-6 secretion in the ATCC2001 strain initially dropped before gradually increasing (Fig. 2A and B), while GM-CSF levels showed a rise, followed by a drop, peaking at 12 and 48 hours (*P < 0.05*, Fig. 2E and F). At 48 hours, GM-CSF levels were significantly higher than those in the itraconazole, amphotericin B, and no-drug groups (Fig. 2E and F). The IL-6/GM-CSF levels rose markedly at 6 and 24 hours *(P < 0.05*, Fig. 2I and J) but dropped significantly at 12 hours (*P < 0.05*, Fig. 2I and J), demonstrating an initial decline followed by an increase. In the 17K1152 strain, IL-6 secretion gradually increased, peaking at 48 hours (*P < 0.05*, Fig. 2C and D) and having the lowest point at 6 hours (*P < 0.05*, Fig. 2C and D). GM-CSF levels showed a significant increase at 24 hours (*P < 0.05*, Fig. 2G and H), which was higher than in other groups. The IL-6/GM-CSF levels significantly dropped at 24 hours (*P < 0.05*, Fig. 2K and L), exhibiting a pattern of initial rise followed by a decline, which was the opposite of the trend observed in the ATCC2001 strain.

With itraconazole intervention, IL-6 secretion in the ATCC2001 strain remained relatively stable, peaking at 24 hours, significantly higher than in other groups (Fig. 2A and B). GM-CSF levels gradually decreased over time (Fig. 2E and F), while IL-6/GM-CSF levels initially rose slightly before declining (Fig. 2I and J). In the 17K1152 strain, IL-6 secretion slightly declined and then rose slightly before dropping again, peaking at 24 hours (Fig. 2C and D). GM-CSF levels increased and then slightly decreased before rising again, peaking at 48 hours and being higher than in the no-drug group (*P < 0.05*, Fig. 2G and H). IL-6/GM-CSF levels at 24 hours were significantly higher than in other groups (*P < 0.05*, Fig. 2K and L), reflecting an initial rise followed by a decline.

Under amphotericin B intervention, IL-6 secretion in the ATCC2001 strain gradually declined, followed by a brief increase, peaking at 6 and 48 hours (*P < 0.05*, Fig. 2A and B) and having the lowest levels at 12 and 24 hours (*P < 0.05*, Fig. 2A and B). GM-CSF levels showed a rise followed by a decline, with the lowest level occurring at 12 hours (*P < 0.05*, Fig. 2E and F). The IL-6/GM-CSF levels at 12 and 48 hours were significantly higher than in the micafungin and itraconazole groups (*P < 0.05*, Fig. 2I and J), while levels at 24 hours were the lowest and significantly lower than in the other groups (*P < 0.05*, Fig. 2I and J), indicating an initial decline followed by an increase. In the 17K1152 strain, IL-6 secretion peaked at 6 hours (*P < 0.05*, Fig. 2C and D), significantly higher than in other groups. The overall trend showed a rapid decline followed by a gradual rise (Fig. 2C and D). GM-CSF levels followed a similar trend to the itraconazole group but were significantly higher (*P < 0.05*, Fig. 2G and H). IL-6/GM-CSF levels were the lowest at 12 and 48 hours (*P < 0.05*, Fig. 2K and L), demonstrating an overall downward trend.

## DISCUSSION

Macrophages, presented on mucosal surfaces and in almost all tissues, play the crucial roles to defend against *C. glabrata*. The interaction between macrophages and *C. glabrata* is mediated by pattern recognition receptors on host cells that recognize pathogen-associated molecular patterns on the fungal surface. Additionally, opsonin receptors recognize opsonized microbes (19).

In this study, electron microscopy observations demonstrated that macrophages effectively engulfed *C. glabrata*. Without antifungal intervention, yeast cells remained relatively intact within macrophages, but macrophage damage worsened over time. In contrast, under micafungin intervention, the cell wall and membrane of *C. glabrata* were severely damaged post-phagocytosis, resulting in significant cytoplasmic damage and a marked reduction in the number of yeast cells within the macrophages. Itraconazole intervention had a moderate impact on the survival of yeast within macrophages, leading to membrane shrinkage; however, some fungal cells were still able to bud and proliferate inside the macrophages. Under amphotericin B intervention, while cell membranes of *C. glabrata* shrank, the fungus remained relatively intact inside macrophages, and macrophage damage increased over time. Overall, amphotericin B had a relatively weak effect in reducing the survival and proliferation of *C. glabrata* inside macrophages. Thus, micafungin appears to be the preferred antifungal agent against engulfed yeast cells, followed by itraconazole, while amphotericin B is not recommended clinically due to its harmful effects on macrophages.

When macrophages engulf *C. glabrata*, the yeast’s cytoplasmic density decreases, especially in standard strains. Compared to standard strains, clinical strains showed more active macrophage exocytosis and more noticeable budding post-phagocytosis. This suggests that clinical strains can continue to replicate within macrophages, with macrophage exocytosis facilitating their evasion from macrophage killing, thereby highlighting the superior adaptability and evasion mechanisms of clinical strains.

Macrophages recognize *Candida* cell wall components, triggering intracellular signaling pathways. The accessibility of these cell wall components (e.g., glucan and/or chitin) is critical for immune recognition (19–21). When the host immune system recognizes β-glucan or chitin in the *Candida* cell wall (20), the fungus is quickly captured by phagosomes, triggering phagocytosis, inducing pro-inflammatory cytokine (e.g., IL-6, GM-CSF), and elevating the production of reactive oxygen and nitrogen species and antimicrobial peptides (20, 22). These antimicrobial defense mechanisms play a crucial role in pathogen elimination (23). In our study, differences in phagocytic response were observed between the clinical and ATCC strains of *C. glabrata*. While the clinical strain appeared to induce more exocytosis and exhibited signs of intracellular persistence, these observations are based on a single clinical isolate and may not be broadly generalizable. Therefore, additional studies using a larger number of clinical strains are needed to validate whether such features represent a consistent advantage in adaptability or immune evasion. Cytokines are essential not only as mediators of the immune system but also in determining the outcome of fungal infections. Pro-inflammatory cytokines like IL-6, IL-8, and TNF-α promote anti-*Candida* effector functions, including regulating leukocyte trafficking, promoting immune cell proliferation, and activating oxidative and non-oxidative metabolic responses in immune cells. GM-CSF effectively activates macrophages, promoting progenitor cell differentiation and recruiting macrophages to infection sites. Given *C. glabrata*’s ability to survive and replicate within macrophages, it may exploit this mechanism to evade immune attacks. However, excessive macrophage recruitment could be detrimental to the host. Therefore, while GM-CSF production and macrophage activation help control infection in the early stages, *C. glabrata* may utilize this mechanism as part of its immune evasion strategy during chronic infections (17). As a limitation, only two cytokines (IL-6 and GM-CSF) were measured in the immune response assessment. The inclusion of additional cytokines such as IL-8, TNF-α, or IL-1β would provide a more comprehensive understanding of the host response.

Without antifungal intervention, macrophages exhibited a relatively weak immune response to the phagocytosis of *C. glabrata*. However, the elevated/reduced cytokine secretion observed in response to different antifungal drugs led us to hypothesize that these drugs may influence the cytokine secretion, thereby affecting the survival or elimination of *C. glabrata* within macrophages. The short-term increase in IL-6 may reflect initial immune activation; however, its subsequent decline suggests that the immune response was insufficient to effectively eliminate the fungus or control its internal proliferation. Additionally, the low levels of GM-CSF might impair macrophage regeneration and repair capacity, hindering effective handling of intracellular damage handling and sustained fungal clearance. Consequently, the lack of effective immune activation in the no-intervention group could result in prolonged fungal survival within macrophages, leading to an increased intracellular fungal burden.

Under micafungin intervention, macrophages displayed a robust immune response to *C. glabrata* phagocytosis, with increasing IL-6 levels indicating enhanced immune activity, possibly due to micafungin-induced fungal damage. High GM-CSF levels likely promote macrophage regeneration and repair, supporting sustained fungal clearance. Thus, micafungin appears effective in reducing fungal survival by boosting immune response and repair mechanisms. In contrast, itraconazole treatment led to a relatively stable immune response, with a stable immune response, with steady IL-6 levels reflecting inhibited fungal proliferation and reduced inflammatory response. However, decreased GM-CSF levels might weaken phagocytic capabilities, extending fungal survival within macrophages, suggesting that itraconazole may impair macrophage function and intracellular fungal clearance. Amphotericin B treatment resulted in an intense immune response and significant cell damage during *C. glabrata* phagocytosis. The rapid decline in IL-6 levels indicates strong toxic effects or cell damage, hindering sustained immune activity against the fungus. The parallel decrease in GM-CSF levels with IL-6 further suggests the impaired macrophage function, potentially promoting fungal survival and replication inside macrophages. Combining observations of structural changes in internalized yeast cells with or without antifungal treatment via TEM, we propose that alterations in immunostimulatory components on the *C. glabrata* cell wall or membrane may reduce its ability to induce macrophage aggregation. This impairment could hinder its immune evasion capabilities, leading to immune destruction by host cells.

The drug susceptibility tests for *C. glabrata* revealed that the 17K1152 strain was less sensitive to three commonly used antifungal drugs than the ATCC2001 strain, with sensitivity to amphotericin B nearing resistance levels. Our findings suggest that polyene drugs may facilitate the replication and survival of clinical strains within macrophages, whereas echinocandins could disrupt the replication, repair, and survival of *C. glabrata* within host cells. TEM showed that, at 6 hours post-phagocytosis, internalized yeast in the amphotericin B group exhibited greater damage than in the micafungin group. However, at 24 hours, micafungin-treated yeast showed greater damage than those treated with amphotericin B. Over time, IL-6 secretion decreased in the amphotericin B group while rising in the micafungin group from 6 to 48 hours. GM-CSF secretion remained consistently higher in the amphotericin B group, with a transient increase at 24 hours in the micafungin group. These results suggest that pro-inflammatory cytokine production decreases in macrophages phagocytosing amphotericin B-pretreated clinical strains, allowing for repair damage and yeast replication within macrophages. Conversely, micafungin treatment led to sustained pro-inflammatory cytokine production, enhancing macrophage immune-killing ability against *C. glabrata* and preventing its persistence. When phagocytosed untreated, *C. glabrata* replicated extensively within the macrophages, triggering active “exocytosis,” with standard strains showing higher replication activity than clinical strains. This highlights the role of macrophages as a reservoir for the persistence, replication, and immune evasion of *C. glabrata*.

## Data Availability

The sequence data for the two *Candida albicans* strains used in this study are publicly available in the NCBI database under the following accession numbers: KU052054.1 (strain 17K1152) and KP674677.1 (strain ATCC2001). All other datasets generated or analyzed during the current study are available from the corresponding author upon reasonable request.
